# Genetic Analysis Based on Mitochondrial *nad2* Gene Reveals a Recent Population Expansion of the Invasive Mussel, *Mytella strigata*, in China

**DOI:** 10.3390/genes14112038

**Published:** 2023-11-03

**Authors:** Shaojing Yan, Peizhen Ma, Chenxia Zuo, Yi Zhu, Xiaojie Ma, Zhen Zhang

**Affiliations:** 1 Laboratory of Marine Organism Taxonomy & Phylogeny, Qingdao Key Laboratory of Marine Biodiversity and Conservation, Institute of Oceanology, Chinese Academy of Sciences, Qingdao 266071, China; yansj@qdio.ac.cn (S.Y.);; 2 State Key Laboratory of Mariculture Biobreeding and Sustainable Goods, Yellow Sea Fisheries Research Institute, Chinese Academy of Fishery Sciences, Qingdao 266071, China; 3 College of Life Sciences, Qingdao University, Qingdao 266000, China; 4 University of Chinese Academy of Sciences, Beijing 100049, China

**Keywords:** alien species, *Mytella strigata*, biological invasion, *nad2*, population genetics

## Abstract

*Mytella strigata* is a highly adaptable invasive alien species that has been established in coastal China since 2014. Mitochondrial DNA (mtDNA) is an important tool for studying the evolution and population genetics of invasive species. In this study, the mitochondrial genome of *M. strigata* from China was sequenced by Illumina high-throughput sequencing and characterized with 13 protein-coding genes (PCGs). By assessing the selective pressure of 13 PCGs, the *nad2* gene had the fastest evolutionary rate and was finally selected for population genetic analysis. A total of 285 *nad2* sequences from seven *M. strigata* populations in China were analyzed and showed obviously T-rich and C-rich characteristics. According to population genetic diversity analysis, all the seven populations had haplotype (gene) diversity (*Hd*) ≥ 0.5 and nucleotide diversity (*Pi*) < 0.005. Haplotype networks showed a “star” distribution. Population historical dynamic analyses showed that Fu’s Fs and Tajima’s D values of all populations were negative except the Qukou (QK) and Beihai (BH) populations. The Zhangzhou (ZJ) and Xiamen (XM) populations were unimodal while the other populations were multimodal. These results suggested that the population of *M. strigata* in China may have passed the bottleneck period and is currently in a state of population expansion.

## 1. Introduction

Along with the global shipping industry’s vigorous development, invasive marine species are more likely to be introduced into new environments where they begin to establish a population [1]. The establishment and spread of invasive species in new areas could have wide-ranging ecological consequences [2]. At fine scales, invasive species may affect changes in the local gene pool [3]. At larger scales, they may affect the structure, composition, diversity, and biological interactions of the recipient communities [4]. Mollusks and crustaceans have the largest number among marine invasive species [5]. *Mytella strigata* (Hanley, 1843) (=*Mytella charruana* (d’Orbigny, 1846)), a marine invasive bivalve [6] native to the Atlantic coast of South America, has been found in many east and southeast Asian countries and regions [7,8]. Studies have shown that *M. strigata* is mainly transmitted over long distances through human activities such as ships’ ballast water, while ocean currents and coastal currents promote its spread in a small range [9]. Its strong adhesion and ability to survive in high densities enable its attachment to the walls and drainage systems of aquaculture ponds, hulls of boats, bottom sediment, and riverbanks of estuaries [10]. Moreover, it poses a serious competitive threat to native species such as *Perna viridis* (Linnaeus,1758) [11]. The ecological and social impacts of invasive species can be significant, but their management is often difficult [12]. At the beginning of an invasion, the genetic diversity of an alien species will generally decrease as they need to adapt to the local environment. This stage is called the bottleneck effect. Once the bottleneck is crossed, the genetic diversity of invasive species will increase and their populations will continuously expand [13]. This covert process can lead to an underestimation of invasive species and limit the effectiveness of management [14]. Therefore, studying population genetic structure is very important to understand the population status of invasive species and strengthen relevant control.

Mitochondrial DNA (mtDNA) is an important tool in researching evolution and population genetics of invasive species due to its relatively high mutation rate and apparent simplicity of maternal inheritance [15]. In most eukaryotes, mitochondrial inheritance usually follows strict maternal inheritance (SMI) [16]. However, a unique doubly uniparental inheritance pattern (DUI) has been reported in more than 100 species of bivalves [17]. Mitochondrial DUI is described as follows: the eggs and spermatozoa contain female-lineage mtDNA (F-type) and male-lineage mtDNA (M-type), respectively. Therefore, the zygote is heteroblastic. If the zygote develops into a female, the animal will be homogenous with the F-type in germ cells and somatic tissues [18]. Otherwise, if the zygote develops into a male, it will be heterogeneous with the M-type in germ cells and F-type in somatic tissues [17]. However, a new inheritance type, namely, atypical DUI, has emerged. The M-type is not only found in male germ cells but also in somatic tissues, such as *Ruditapes philippinarum* (Adams & Reeve, 1850) [19] and *Mytilus galloprovincialis* (Lamarck, 1819) [20]. In previous studies, *M. strigata* were also shown to possess atypical DUI, as M-COI was found in the adductor muscle [21,22]. Because the two haplotypes in DUI species are related to sex, the evolution rate of the haplotypes may be different, which may affect the accuracy of population genetic analysis [7,22]. At the same time, population genetic analysis also relies on the relatively high mutation of mitochondrial gene makers. In general, genes with less selective pressure are thought to have more mutations [23] and as a result, the ratios of the nonsynonymous substitution rate (Ka) and synonymous substitution rate (Ks) were commonly employed. Together, it is necessary for more suitable mitochondrial gene makers to be screened and employed for population genetic analysis. 

Here, Illumina high-throughput sequencing was performed on the mitochondrial genome of *M. strigata* from China. Selective pressure was analyzed on a mitochondrial protein-coding gene (PCG) to choose the best mitochondrial marker for population genetic analysis. Then, the population genetic diversity and population history dynamics of seven populations of *M. strigata* in coastal China were carried out. The aim of this study was to (1) select more suitable mitochondrial PCGs for population genetic analysis, (2) evaluate the invasive status of *M. strigata* populations in coastal areas of China, and (3) provide molecular biological data for assessing the expansion and determine the need for control measures of *M. strigata*.

## 2. Materials and Methods

### 2.1. Mussels and Sampling

**Figure 1:**
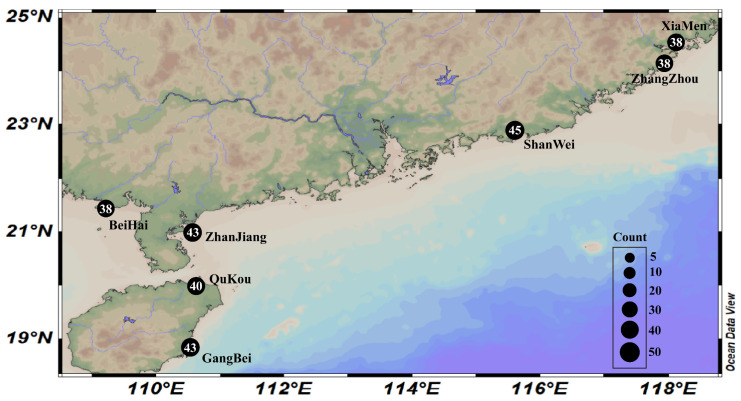
Sampling station maps for the seven populations of *Mytella strigata*. Black circles represent sampling points. The size of the black circle indicates the number of samples.

All experiments were performed in accordance with the relevant guidelines and regulations. In April 2023, a total of 252 individuals were collected from seven populations in China: Zhanjiang (ZJ), Beihai (BH), Shanwei (SW), Zhangzhou (ZZ), Qukou (QK), Gangbei (GB), and Xiamen (XM) (Figure 1, Table 1). All the samples were directly collected from the attachment substrates, including rocks, concrete, oyster shells, and net cages, in intertidal zones. All samples were identified as *M. strigata* according to the morphological descriptions (Figure S1) [10,11,24]. All samples were preserved in 95% alcohol and deposited in the Laboratory of Marine Organism Taxonomy and Phylogeny, Qingdao Key Laboratory of Marine Biodiversity and Conservation, Institute of Oceanology, Chinese Academy of Sciences, Qingdao, China.

### 2.2. DNA Extraction, Sequencing, Assembly, and Annotation for Mitochondrial Genomes

One sample from ZJ was chosen for mitochondrial genome sequencing. Total DNA was extracted from the adductor muscle using a TIANamp Marine Animals DNA kit (DP324-03, Tiangen Biotech Co., Ltd., Beijing, China) according to the reagent instructions. The genomic library was constructed with the whole-genome shotgun approach and sequenced on the Illumina NovaSeq platform (Illumina, San Diego, CA, USA) at Shanghai Personal Biotechnology Co., Ltd. using the 2 × 150 bp paired-end sequencing mode. A5-miseq v20150522 software [25] and SPAdes v3.9.0 software [26] were used for the de novo assembly of high-quality data. The complete mitogenome sequences were uploaded to the MITOS2 web server for functional annotation [27]. The genetic code selection was set to “Invertebrate” and the other settings were adjusted according to the default parameters. The boundaries of PCGs were determined by an ORF finder (https://www.ncbi.nlm.nih.gov/orffinder, accessed on 13 October 2023), and then they were corrected manually and annotated by comparison with the previous mitochondrial genomes of *M. strigata* (Genbank accession nos.: MT800514 and MT991018).

### 2.3. Selective Pressure (Ka/Ks) Analysis

PhyloSuite v1.2.2 software was employed to perform the preparation of the PCG sequences from mitochondrial genomes sequenced in this study [28]. The remaining two mitochondrial genomes used for selective pressure analysis came from GenBank (GenBank accession nos.: MT800514 and MT991018). Multiple Alignment Using Fast Fourier Transformation (MAFFT v7.505) software was used to align PCGs independently [29]. The codon mode for PCGs was selected using the invertebrate mitochondrial code table. Gblocks was employed here to conduct the initial alignments and retain only the conserved regions with clear positional homology [30]. DnaSP v6.12.03 software [31] was then used to calculate the Ka and Ks of each PCG from mitochondrial genomes of *M. strigata*. Based on the selective pressure results, the PCG with highest variation, *nad2*, was selected as the marker gene for genetic analysis.

### 2.4. Amplification and Sequencing of Marker Gene

The total DNA of individuals from all seven populations was extracted from the adductor muscle using a TIANamp Marine Animals DNA kit (DP324-03, Tiangen Biotech Co., Ltd., Beijing, China). Then, PCR primers were designed using primer premier v5.5.0 software for the mitochondrial *nad2* gene: forward primer 5′-GGTGATAAGAATAATAATAAGACCTTTG-3′, reverse primer 5′-CCACTCTTATGACCACTATTATC-3′. The PCR reaction mixture contained 0.5 µL of each primer, 1.0 µL of DNA, 12.5 µL of 2× Taq PCR MasterMix (PC1120; Beijing Solarbio Science & Technology Co., Ltd., Beijing, China), and 10.5 µL of ddH_2_O in a total volume of 25 µL. The PCR procedure involved initial denaturation at 95 °C for 3 min; 32 cycles of 95 °C for 30 s, 48 °C for 1 min, and 72 °C for 1 min; and a final extension at 72 °C for 5 min. Finally, agarose gel electrophoresis was used to detect the purity of PCR products and the Applied Biosystems 3730xl Genetic Analyzer (Beijing Tsingke Biotech Co., Ltd., Beijing, China) was used for sequencing.

### 2.5. Data Analysis

MEGA v7.0.26 software was employed to analyze the base composition and sequence variation of the *nad2* gene sequence. DnaSP v6.12.03 [31] software was used to analyze the number of haplotypes (*h*), number of polymorphic (segregating) sites (*S*), haplotype (gene) diversity (*Hd*), nucleotide diversity (*Pi*), and the average number of nucleotide differences (*K*) of *nad2* sequences from the seven populations. MEGA v7.0.26 software was used to align and evaluate the inter- and intra-population genetic distances of *nad2* sequences from the seven populations with the Maximum Composite Likelihood model. The software of Arlequin v3.5 was used to perform the analysis of molecular variance (AMOVA) of the populations. PopART v1.7 [32] and Arlequin v3.5 [33] were used to construct haplotype networks. The genetic differentiation coefficient (F-statistics, *FST*) of inter-population and its significance, as well as Tajima’s D and Fu’s Fs, were also calculated using Arlequin v3.5 [33]. Finally, DnaSP v6.12.03 [31] software was used to perform mismatch distribution.

## 3. Results

### 3.1. Mitochondrial Genome of M. strigata

The mitochondrial genome measured in this study had a double-stranded circular structure, with GenBank accession No. OR666116. The mitochondrial genome consisted of 2 rRNA genes (*12s* and *16s*), 22 tRNA genes, and 13 PCGs, including *atp6*, *cox1*, *cox2*, *cox3*, *cytb*, *nad1*, *nad2*, *nad3*, *nad4*, *nad4l*, *nad5*, *nad6*, and *atp8*.

The results of selective pressure analysis showed that all the PCGs had Ka/Ks < 1 (Table 2). This means that these genes exhibit a purifying selection. Among them, the selective pressure of *nad2* was Ka/Ks = 0.15321, the maximum of these genes.

### 3.2. Base Composition and Sequence Variation of nad2

A total of 285 *nad2* sequences were obtained from seven populations in this study and the sequence length was 691 bp. The percent identity of these sequences was found to be as high as 99.71% by aligning with *M. strigata* sequences in GenBank. The average contents of A, T, C, and G of the *nad2* sequence were 13.67%, 34.88%, 41.44%, and 10.02%, respectively, exhibiting obviously T-rich and C-rich characteristics (Table 3). Totally, 31 variable sites were detected, accounting for 4.49% of the total number of sites. Among them, there were 18 parsimony informative sites and 13 singleton variable sites. In addition, there were two transition sites and one transversion site in mitochondrial *nad2* sequences, and the average transition to transversion ratio was 3.9.

### 3.3. Population Genetic Diversity Analysis

All 285 *nad2* gene sequences were used for genetic diversity analysis. Results showed that a total of 31 haplotypes (GenBank accession Nos. OR604589-OR604619) and 31 polymorphic (segregating) sites were charactered (Table 4). Among them, the ZJ population (19 *h* and 19 *S*) had the most haplotypes and polymorphic (segregating) sites, while the BH population (7 *h*) and QK population (9 *S*) had the fewest haplotypes and polymorphic (segregating) sites, respectively. The *Hd*, *Pi,* and *K* of all populations were 0.869, 0.00384, and 2.652, respectively. Similarly, the ZJ population had the highest *Hd* (0.898), *Pi* (0.00403), and *K* (2.783). The difference was that the BH population had the lowest *Pi* (0.00301) and *K* (2.077), while the GB population occupied the lowest *Hd* (0.712).

The population genetic distance and genetic differentiation analysis of *M. strigata* is shown in Table 5. The genetic distances between populations ranged from 0.00328 (BH and XM) to 0.00467 (ZZ and GB). And within populations, the BH population had the smallest genetic distance of 0.00303 and the ZJ population had the largest one of 0.00407. In addition, larger genetic differentiation (0.15~0.25) was observed at GB-ZJ (0.16360), GB-BH (0.15309), GB-SW (0.17637), and GB-ZZ (0.22865) (*p* < 0.05). In QK-ZJ (0.07061), QK-BH (0.05826), QK-SW (0.07391), QK-GB (0.13784), and GB-XM (0.14683), there was a moderate genetic differentiation (0.05~0.15) (*p* < 0.05). The results of AMOVA (Table 6) showed that 7.23% of the genetic variation occurred among populations and 92.77% occurred within populations.

**Figure 2:**
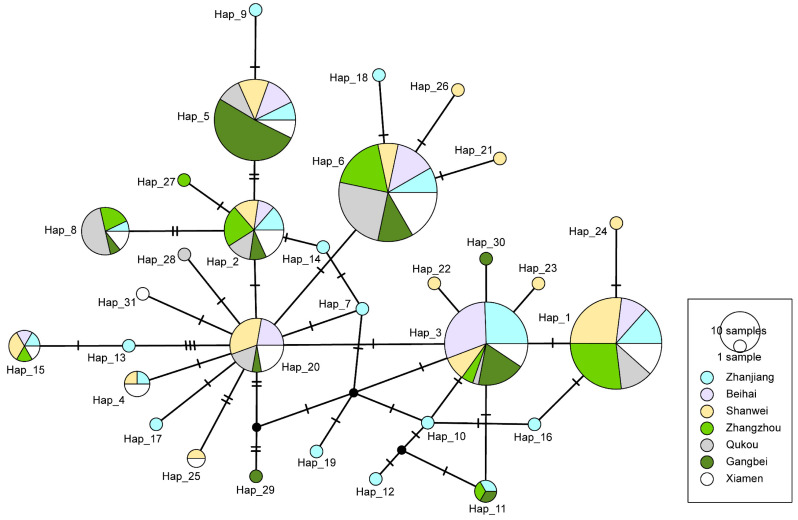
Haplotype TCS networks in seven populations of *Mytella strigata* based on mitochondrial *nad2* gene. The size of the circle represents the frequency of the haplotype. Different colors represent different populations. The number of segments of the connecting lines between haplotypes represents the number of nucleotide replacements.

The haplotype network based on the *nad2* gene showed a “star” distribution with Hap_20 as the center (Figure 2). Among the 31 haplotypes, Hap_1, Hap_3, Hap_5, and Hap_6 were the dominant haplotypes with 196 individuals. Hap_2, Hap_3, and Hap_6 were shared by all the seven populations. Of all 20 unique haplotypes, the ZJ population possessed 10 haplotypes (Hap_16~Hap_19), while the remaining populations had another 10 haplotypes (Hap_11, Hap_12, and Hap_14) except the BH population. Significantly, the ZJ population had the most diverse and numerous haplotypes (19 and 43, respectively).

### 3.4. Neutrality Test and Mismatch Distribution Analysis

**Figure 3:**
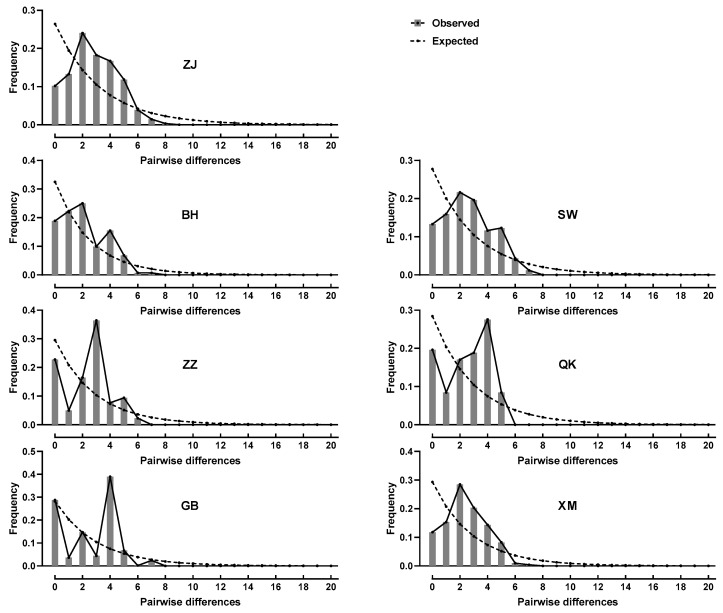
Mismatch distribution of *Mytella strigata* populations. The bar chart represents the distribution observed, and the line chart represents the distribution predicted by the model. ZJ: ZhangJiang, BH: Beihai, SW: Shanwei, ZZ: Zhangzhou, QK: Qukou, GB: Gangbei, XM: Xiamen.

The results of the neutrality test (Table 7) showed that the Fu’s Fs values of all populations except the BH and QK populations were negative. The Fu’s Fs tests of the ZJ and SW populations were statistically significant (*p* < 0.05) and the mismatch distribution analysis (Figure 3) showed that the ZJ population displayed a unimodal distribution. The XM population also presented as a unimodal distribution but there was no statistical significance (*p* > 0.05). The mismatch distribution of the BH, ZZ, QK, and GB populations showed bimodal or multimodal distribution. In addition, the Tajima’s D values of the QK population were positive, while those of other populations were negative. There were no statistically significant values of Tajima’s D test in all populations (*p* > 0.05).

## 4. Discussion

### 4.1. Screening and Characterization of the Molecular Marker

Compared to the more conserved nuclear genome, the mitochondrial genome has a faster rate of genetic mutation. In primates and mollusks, the mutation rate of mtDNA genomes is about 5–10 times and 4 times than nuclear DNA, respectively [34,35]. In bivalves, although the ratio of the mtDNA to nuclear DNA mutation rate is lower (about 1.8), it is still higher than nuclear DNA [35]. At the same time, studies have shown that the mitochondrial mutation rate of Pteriomorphia, which *M. strigata* belongs to, is faster than that of other infraclasses in bivalves [36]. On the single gene level, the commonly used mitochondrial genes such as *cox1* tend to have more genetic diversity than the nuclear genes such as *its1* [22,37,38]. Due to the high rate of evolution, mtDNA could be a useful molecule for the analysis of evolutionary processes [39]. Therefore, mitochondrial genome analysis was performed on *M. strigata* taken from coastal areas of China and 13 PCGs were identified. Selective pressure analysis showed that the selective pressure of all 13 PCGs was Ka/Ks < 1, indicating that all the PCGs are subject to purifying selection. Purifying selection acts to weed out harmful mutations from the population and helps maintain the fitness of the species [40]. The maximum selective pressure (Ka/Ks = 0.15321) was found in *nad2*, suggesting that *nad2* is subject to the least selective pressure and has the highest variation. Therefore, the *nad2* gene was selected as the most appropriate genetic marker to analyze the population genetics of *M. strigata*. The only molecular marker used in the previous population genetic analysis of *M. strigata* was *cox1* [7,21,22]. However, in contrast to *cox1*, the *nad2* gene has no sex-related haplogroup, which is the second reason it was chosen.

In this study, the A + T base content was slightly lower than the G + C base content in the mitochondrial *nad2* gene of *M. strigata*. The result showed a CG bias. Base bias is a characteristic of sequence evolution, which may result from natural mutation [41]. Although high AT base content is more common in invertebrate mtDNA [42], the nucleotide mutation rate of different fragments is inconsistent, which leads to different GC content in different parts [43]. Meanwhile, the difference in base content is species-specific [44]. In general, transition is more likely to occur than transversion because transition does not change the type of base [45]. In the results, the average transition to transversion ratio was 4.6. This means that the sequence variation of *nad2* occurs mostly between a purine and a purine or between a pyrimidine and a pyrimidine.

### 4.2. Population Genetic Diversity and Structure of M. strigata

In the results, all the seven populations had *Hd* ≥ 0.5 and *Pi* < 0.005, suggesting that *M. strigata* has crossed the bottleneck effect in coastal China [46]. It is currently undergoing a population expansion with a rapid population growth and accumulation of mutations [47]. The invasion of *M. strigata* in China began around 2014 [7]. Zuo et al. hypothesized that *M. strigata* in China had experienced or was currently experiencing a population bottleneck during 2020~2021 [22], and this study further proves that the Chinese population of *M. strigata* in 2023 is in the stage of population expansion after the bottleneck effect. The phenomenon of the population expansion of *M. strigata* in China could also be seen in practical production. In 2019, a survey revealed that *M. strigata* has been present in farming ponds of *Meretrix petechialis* (Lamarck, 1818) in Taiwan and caused serious impact [10]. In 2020, *M. strigata* was found attached to an oyster farming facility in Hainan [7]. In 2022, the density of *M. strigata* surpassed that of the native species *P. viridis* in Donghai island [9]. In addition, the ZJ population, where *M. strigata* was first found in mainland China, has the highest genetic diversity. This corresponds to the greatest genetic distance within the ZJ population

Based on *nad2* sequences, the genetic distance between populations of *M. strigata* ranged from 0.00328 to 0.00467. The genetic distance between the BH population and the XM population was the nearest, indicating a closer relationship [48], while that of the ZZ population and the GB population was the furthest. AMOVA analysis results showed that the genetic variation of *M. strigata* was mainly from within the population rather than between the populations. This was also evidenced by genetic differentiation. It is generally believed that genetic differentiation between populations is small, moderate, and larger when *F_ST_* is 0~0.05, 0.15~0.25, and 0.15~0.25, respectively [49]. Except for the QK and GB populations, the genetic differentiation among other populations was not obvious. This may be related to the geographical location of the QK and GB populations on Hainan Island. In addition, it is found that Hap_20 acts as the center and diverges to other haplotypes according to the haplotype network. Therefore, Hap_20 is presumed to be an ancient haplotype [50]. The “star” distribution of haplotype networks also supported that the different populations of *M. strigata* began to expand after experiencing bottleneck [51]. However, the ZJ population and the ZZ population do not exist in the ancient haplotype Hap_20. From this, we hypothesize that the ZJ population and the ZZ population may have been introduced by other populations. This might also explain why there was a large genetic distance within the ZJ population and a small genetic distance between the ZJ population and other populations. The ZJ and SW populations had the most unique haplotypes, indicating that these two populations could have undergone adaptive evolution [7].

### 4.3. Population Historical Dynamics of M. strigata

Neutrality tests and mismatch distribution are two effective tests to study the historical dynamics of populations [52]. A population is identified as stable when the value of the neutrality test approaches 0. In another case, a population is considered to have expanded in history when the value is negative and statistically significant [53]. In our results, Fu’s Fs and Tajima’s D values of all populations except the QK and BH populations were negative. However, only the ZJ population and the SW population had statistically significant Fu’s Fs values. Tajima’s D test is more likely to reveal the history of expansion of an ancient population, while Fu’s Fs test is more sensitive to the recent expansion of a population [54,55]. Therefore, it is speculated that there have been recent population expansion events in the ZJ population and the SW population. For a stable population, mismatch distribution analyses will display bimodal or multimodal distribution. However, if a population has experienced expansion or sustained growth, the results will show a unimodal distribution. According to the results, the ZJ and XM populations showed a unimodal distribution, but the neutrality test of the XM population was not significant. This once again supported that recent expansion and sustained growth had occurred in the ZJ population [56].

## 5. Conclusions

In this study, gene *nad2* was ultimately selected as the best mitochondrial marker for population genetic analysis by assessing the selective pressure of 13 mitochondrial PCGs. Population genetic analysis showed that the seven *M. strigata* populations in China could be expanding after the bottleneck. Among them, recent expansion and sustained growth events had occurred in the ZJ population. Therefore, relevant measures, including removal (physical removal and biological control) and utilization (human food and feed), need to be taken to prevent the formation of large and stable invasive populations of *M. strigata* in the future.

## Figures and Tables

**Table 1:** Information about the sampling stations. ZJ: ZhangJiang, BH: Beihai, SW: Shanwei, ZZ: Zhangzhou, QK: Qukou, GB: Gangbei, XM: Xiamen.

Location	Collection Date	Geographical Coordinates	Quantity
ZJ	2 April 2023	110°31′43″ E, 20°55′53″ N	43
BH	3 April 2023	109°11′36″ E, 21°24′42″ N	38
SW	5 April 2023	115°36′28″ E, 22°51′25″ N	45
ZZ	6 April 2023	117°58′0″ E, 24°9′14″ N	38
QK	1 April 2023	110°35′7″ E, 19°59′7″ N	40
GB	1 April 2023	110°30′50″ E, 18°53′12″ N	43
XM	6 April 2023	118°4′41″ E, 24°28′1″ N	38
Total		-	285

**Table 2:** The evolutionary constraint (Ka/Ks) analyses of 13 mitochondrial protein-coding genes (PCGs). Ka: nonsynonymous substitution rate, Ks: synonymous substitution rate calculations.

Mitochondrial PCGs	Ka	Ks	Ka/Ks
*atp6*	0.00126	0.01233	0.10219
*atp8*	0.00000	0.03750	0.00000
*cox1*	0.00113	0.01614	0.07001
*cox2*	0.00279	0.00000	-
*cox3*	0.00000	0.01351	0.00000
*cytb*	0.00000	0.00375	0.00000
*nad1*	0.00000	0.01453	0.00000
*nad2*	0.00093	0.00607	0.15321
*nad3*	0.00000	0.01633	0.00000
*nad4*	0.00134	0.01938	0.06914
*nad4l*	0.00000	0.00000	-
*nad5*	0.00202	0.01650	0.12242
*nad6*	0.00187	0.01744	0.10722

**Table 3:** Base composition of *nad2* gene sequence by population (%). ZJ: ZhangJiang, BH: Beihai, SW: Shanwei, ZZ: Zhangzhou, QK: Qukou, GB: Gangbei, XM: Xiamen. A: Adenine, T: Thymine, C: Cytosine, G: Guanine.

Nucleotide Composition	A	T	C	G
ZJ	13.50	34.66	41.26	10.58
BH	13.33	35.31	41.51	9.85
SW	13.19	35.56	41.08	10.18
ZZ	13.50	35.40	41.26	9.85
QK	13.95	34.03	42.26	9.76
GB	14.78	34.66	40.74	9.83
XM	13.41	34.55	41.94	10.10
Average	13.67	34.88	41.44	10.02

**Table 4:** Genetic diversity analysis based on *nad2* molecular markers. ZJ: ZhangJiang, BH: Beihai, SW: Shanwei, ZZ: Zhangzhou, QK: Qukou, GB: Gangbei, XM: Xiamen. *h*, number of haplotypes; *S*, number of polymorphic (segregating) sites; *Hd*, haplotype (gene) diversity; *Pi*, nucleotide diversity; *K*, average number of nucleotide differences.

	*h*	*S*	*Hd*	*Pi*	*K*
ZJ	19	19	0.898	0.00403	2.783
BH	7	10	0.811	0.00301	2.077
SW	14	18	0.867	0.00377	2.606
ZZ	8	11	0.772	0.00345	2.385
QK	8	9	0.804	0.00364	2.517
GB	9	13	0.712	0.00368	2.545
XM	11	15	0.882	0.00349	2.410
Total	31	31	0.869	0.00384	2.652

**Table 5:** Genetic distance (diagonal and below diagonal) and genetic differentiation coefficient (F-statistics, *F_ST_*) (above diagonal) between populations of *Mytella strigata* based on *nad2* gene sequences. ZJ: ZhangJiang, BH: Beihai, SW: Shanwei, ZZ: Zhangzhou, QK: Qukou, GB: Gangbei, XM: Xiamen. A: Adenine, T: Thymine, C: Cytosine, G: Guanine.

	ZJ	BH	SW	ZZ	QK	GB	XM
ZJ	0.00407	−0.00912	−0.00360	0.01563	0.07061 *	0.16360 *	0.01903
BH	0.00351	0.00303	−0.00192	0.02429	0.05826 *	0.15309 *	0.00331
SW	0.00392	0.00341	0.00380	0.00382	0.07391 *	0.17637 *	0.01665
ZZ	0.00383	0.00333	0.00365	0.00348	0.04562 *	0.22865 *	0.01477
QK	0.00417	0.00356	0.00404	0.00375	0.00367	0.13784 *	0.00103
GB	0.00465	0.00399	0.00457	0.00467	0.00429	0.00372	0.14683 *
XM	0.00386	0.00328	0.00372	0.00355	0.00360	0.00424	0.00351

* indicates significant level *p* < 0.05.

**Table 6:** AMOVA results of *Mytella strigata* among seven populations. *d.f.*: degrees of freedom.

Source of Variation	*d.f.*	Sum of Squares	Variance Components	Percentage of Variation
Among populations	6	31.110	0.09690 Va	7.23
Within populations	278	345.424	1.24253 Vb	92.77

**Table 7:** Fu’s Fs and Tajima’s D test from different populations of *Mytella strigata*. ZJ: ZhangJiang, BH: Beihai, SW: Shanwei, ZZ: Zhangzhou, QK: Qukou, GB: Gangbei, XM: Xiamen.

	Fu’s Fs	*p*-Value	Tajima’s D	*p*-Value
ZJ	−10.53399	0.000 *	−1.18943	0.114
BH	0.05077	0.531	−0.38389	0.410
SW	−4.45261	0.028 *	−1.17457	0.128
ZZ	−0.25569	0.495	−0.27226	0.419
QK	0.01903	0.541	0.55355	0.737
GB	−0.53739	0.439	−0.47109	0.376
XM	−2.58937	0.101	−1.04582	0.167

* indicates significant level *p* < 0.05. Tajima’s D and Fu’s Fs are both neutrality test values.

## Data Availability

Relevant information has been added in the article.

## Supplementary Materials

The following supporting information can be downloaded at https://www.mdpi.com/article/10.3390/genes14112038/s1, Figure S1: Shell morphology of *Mytella strigata*.
